# Massachusetts Veterinary Medical Association

**Published:** 1892-02

**Authors:** Austin Peters

**Affiliations:** Secretary


*Massachusetts Veterinary Association.*—The regular meeting of the Massachusetts Veterinary Association, held at 19 Boylston Place, Boston, Wednesday evening, December 23rd, 1891. President, Dr. L. H. Howard, in the chair. Members present: Drs. Bryden, Blackwood, Howard, Winchester, Harrington and the Secretary; honorary member, Dr. Stickney; guest, Dr. G. P. Dillon. Minutes of previous meeting read and accepted. Dr. Winchester reported for committee on securing the appointment of a veterinary pathologist at the State Agricultural Experiment Station at Amherst, that much work had been done to this end, and that the committee is very much alive.

Applications for membership: Dr. W. A. Hitchcock, of Malden, and Dr. J. M. Parker, of Fitchburg. The secretary presented their credentials for the approval of the executive committee. President Howard, ex-officio member, was the only one of the committee present. He reported favorably upon them, and their names were laid on the table to be voted upon at the next regular meeting.

Dr. Winchester reported a case of prolapsis uteri in a cow, just calved; when he saw the case the uterus was strangulated and black; replacement was impossible, so he ligated the tumor at its base and amputated the uterus and vagina. This was five days previous to the meeting, and during this time the cow has not yet missed a meal, the appetite is good, she suckles her calf, gives a large quantity of milk and feels well.

Dr. Winchester, as a member of the Comitia Minora of the U. S. V. M. A., wished to know the sense of the meeting upon trying to have the next meeting of the U. S. Society in Boston. Dr. Howard thought it would be a good plan to have it here, and also to interest other New England veterinarians in the matter. It was finally decided to discuss the matter more thoroughly at the next meeting of the Association, the Secretary to give notice of the fact in the announcements of the next meeting.

Dr. Bryden showed the hoofs from the hind feet of the old pony that was destroyed with "springhalt," a report of which he read at the last meeting. Meeting then adjourned.

AUSTIN PETERS, *Secretary.*

